# A Prism Vote method for individualized risk prediction of traits in genotype data of Multi-population

**DOI:** 10.1371/journal.pgen.1010443

**Published:** 2022-10-27

**Authors:** Xiaoxuan Xia, Yexian Zhang, Rui Sun, Yingying Wei, Qi Li, Marc Ka Chun Chong, William Ka Kei Wu, Benny Chung-Ying Zee, Hua Tang, Maggie Haitian Wang

**Affiliations:** 1 Centre for Clinical Research and Biostatistics, JC School of Public Health and Primary Care, the Chinese University of Hong Kong, Hong Kong SAR, China; 2 CUHK Shenzhen Research Institute, Shenzhen, China; 3 Department of Statistics, the Chinese University of Hong Kong, Hong Kong SAR, China; 4 The 7th affiliated hospital of Sun Yat-Sen University, Shenzhen, China; 5 Department of Anaesthesia and Intensive Care, the Chinese University of Hong Kong, Hong Kong SAR, China; 6 Department of Genetics, Stanford University, California, United States of America; Genome Institute of Singapore, SINGAPORE

## Abstract

Multi-population cohorts offer unprecedented opportunities for profiling disease risk in large samples, however, heterogeneous risk effects underlying complex traits across populations make integrative prediction challenging. In this study, we propose a novel Bayesian probability framework, the Prism Vote (PV), to construct risk predictions in heterogeneous genetic data. The PV views the trait of an individual as a composite risk from subpopulations, in which stratum-specific predictors can be formed in data of more homogeneous genetic structure. Since each individual is described by a composition of subpopulation memberships, the framework enables individualized risk characterization. Simulations demonstrated that the PV framework applied with alternative prediction methods significantly improved prediction accuracy in mixed and admixed populations. The advantage of PV enlarges as genetic heterogeneity and sample size increase. In two real genome-wide association data consists of multiple populations, we showed that the framework considerably enhanced prediction accuracy of the linear mixed model in five-group cross validations. The proposed method offers a new aspect to analyze individual’s disease risk and improve accuracy for predicting complex traits in genotype data.

## Author summary

In this study, we developed a statistical approach to dissect and predict human complex traits using genotype data. Distinct from existing methods that focus on refining effect size of genetic factors, the proposed method, Prism Vote, improves risk prediction from the dimension of individual, such that disease probability of a subject is regarded as a composite risk shaded from multiple subpopulations, thereby drawing information from both stratum-specific estimation and individualized risk composition. We showed in simulation studies that the PV enhanced prediction performance of several base prediction models significantly, particularly when genetic heterogeneity in the data is high. We also demonstrated in real genome-wide association study data of mixed populations that the PV considerably enhanced prediction accuracy of linear mixed models for traits including body-mass index, height, hypertension, and others. The PV framework offers an effective and scalable approach to leverage subpopulation information to perform risk prediction in mixed populations.

## Introduction

Genome-wide genetic markers encode a sizable portion of common human traits heritability [1]. One attractive application of the susceptible single nucleotide polymorphisms (SNPs) is to construct prediction models for assessing disease risk. Previous association studies have demonstrated that most complex traits possess a polygenic background influenced by collective genetic variants of moderate to small effects [2–4], as exhibited in the human height [2], bipolar disorder [3], and cancers [4]. Due to genetic heterogeneity of complex diseases, a considerable part of the identified risk predisposition loci does not replicate across populations [5]. Currently, near 80% of genetic association studies were conducted in populations of European ancestry [6], and disease risk estimation derived from these datasets alone might not be representative for application in the non-European populations [7,8]. Developing statistical methods for cross-population disease prediction is crucial for improving the genetic risk profiling, precision medicine interventions, and reducing health disparities [9]. Several methods were proposed for trans-ethnic risk prediction. Cai *et al*. improved the Polygenic risk score (PRS)-based prediction for a target minority population by estimating transferrable effect of a common set of SNPs between the target and a larger auxiliary population [10]. Coram *et al*. developed a prediction method for minority population by incorporating risk loci from an auxiliary population as the random component in linear mixed model (LMM) [11]. On the other hand, joint analysis of multiple populations may offer a way to leverage all available samples in the minority groups, generate an integrative risk prediction inference for diverse populations, and in turn facilitate new studies to be carried out in non-European cohorts. However, direct combination of cohorts would render prediction accuracy because of the heterogeneous genetic architecture across population groups, and meta-analysis by mixed models were developed to combine estimations from multiple populations [12,13]. As these methods improve prediction by refining the effect size and SNP subsets in the target population, individuals carrying the same allelic variations at these SNPs would be estimated with the same degree of risk.

Alternatively, we consider prediction in multiple populations by leveraging the dimension of individual identity, which can be incorporated together with the SNP-centered methods to improve risk prediction. Under the proposed framework, named the Prism Vote (PV), the disease risk of a subject can be considered as a composite risk shaded from multiple subpopulation strata, in which stratum-specific genetic risk are characterized, while overall risk of the subject is integrated using Bayesian probability according to one’s propensity to subpopulations. Therefore, subjects with identical alleles at risk loci may be predicted with non-identical disease probability as subpopulation propensity varies. In the PV framework, subpopulation can be regarded as strata of more homogeneous genetic architecture compared to the non-stratified data, which might be shaped by ancestral difference or implying subject groups experiencing similar exposures altering gene-environmental interactions. The prediction utility of PV is demonstrated in three simulation studies and two genome-wide association datasets of mixed populations.

## Methods

### The method overview

The PV leverages on the genetic heterogeneity and polygenicity nature of complex traits. The detection of trait-associated markers, thousands of variants with modest effect size, are sensitive to the underlying genetic architecture of data. Stratification of samples may lead to the identification of stratum-specific risk loci and effects. The framework obtains stratum-wise risk estimates and delivers the individualized risk probability of traits through modelling the disease of a subject as a composite risk outcome from multi-layer subpopulations.

Suppose risk of a trait for subject *i* is attributed from multiple risk strata. Let *Y* denote a phenotype of binary outcome, and ***x***_*i*_ is the genotype matrix of subject *i*. Disease probability of the subject can be written as,

$$\mathrm{Pr}\left(Y=1|x_{i}\right)=\sum_{k=1}^{K}\mathrm{Pr}\left(Y=1|i\in k,x_{i}\right)\;\mathrm{Pr}\left(i\in k|x_{i}\right),$$ (1)

**Fig 1:**
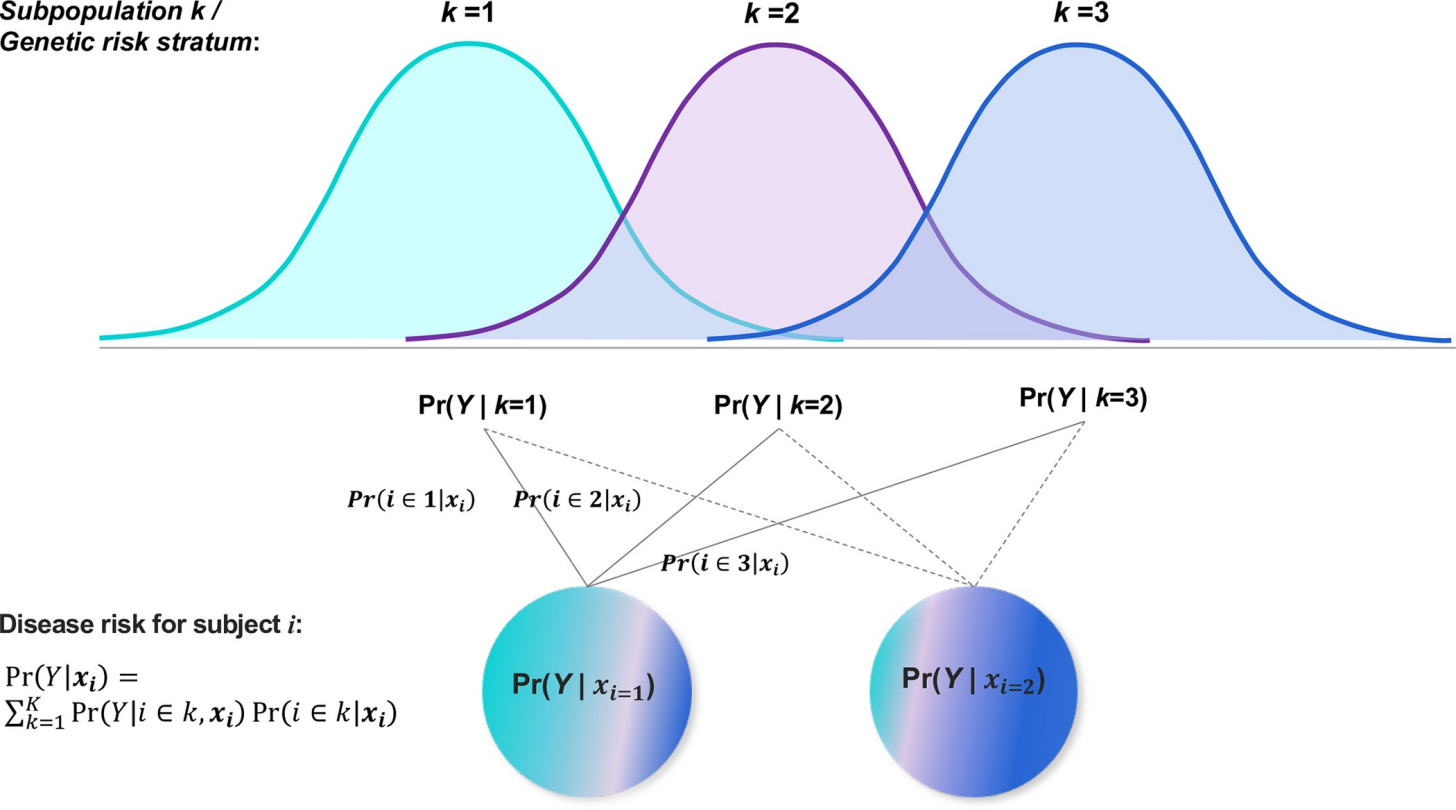
The Prism Vote (PV) framework for individualized risk prediction of traits. The PV views a complex trait of a subject as a composite risk outcome shaded from subpopulation strata, in which stratum-specific risk predictors may be estimated in a subpopulation comparatively more homogeneous. The stratum-wise risk is obtained in subpopulations by Pr(*Y*|*i*, ***x***_*i*_), which are multiplied to a subject’s subpopulation propensity Pr (*i*∈*k*|***x***_*i*_). The total predicted risk of a trait for an individual is the aggregated risk estimate from all subpopulations. The PV framework introduced the dimension of individual identity for modeling disease risk. Hence, the unique spectrum of propensities of each subject to subpopulations offers an individualized risk assessment outcome.

**Eq 1** is referred to as the PV probability of a trait for a subject. It could be generalized to $E\left(Y|x_{i}\right)=\sum_{k=1}^{K}E\left(Y|i\in k,x_{i}\right)\;\mathrm{Pr}\left(i\in k|x_{i}\right)$ for a continuous *Y*. In the equation, $Pr(Y=1|i\in k,x_{i})$ is the disease risk in stratum or subpopulation *k*, to be obtained by a base prediction model; and Pr (*i*∈*k*|***x***_*i*_) is the propensity of subject *i* belonging to stratum *k*, calculated by the Bayes theorem:

$$\mathrm{Pr}\left(i\in k|x_{i}\right)=\frac{Pr\;(i\in k;x_{i})}{Pr\;(x_{i})}=\frac{Pr\;(x_{i}|i\in k)Pr\;(i\in k)}{\sum_{k=1}^{K}Pr\;(x_{i}|i\in k)Pr\;(i\in k)},$$ (2)

in which Pr(***x***_*i*_|*i*∈*k*) is the probability of observing ***x***_*i*_ given subject *i* belongs to stratum *k*∈{1,⋯,*K*}; and Pr(*i*∈*k*) is estimated by the proportion of *k*^*th*^ stratum out of all samples. **Fig 1** shows a schematic diagram of the PV framework. The term “prism” reflects the interpretation that a subject’s disease risk is decomposed into a spectrum of risk distributions by population strata.

### PC-based population stratification and membership estimation

A PC-based approach is adopted to cluster subpopulations as the PCA requires less assumptions and could be applied in various data types. Let g_*ij*_ be genotype of subject *i* for SNP *j*, coded by minor allele counts (0, 1, 2), *i* = 1,⋯,*N*, and *j* = 1,⋯,*P*. The genetic matrix **G**_*N*×*P*_ is normalized to ***X***, by letting $x_{ij}{=(g}_{ij}-{\bar{g}}_{j})/\sqrt{{2p}_{j}(1-p_{j})}$, where ${\bar{g}}_{j}=\frac{\sum_{i=1}^{N}g_{ij}}{N}$ is column mean of SNP vector *j*, and $p_{j}=\left(1+\sum_{i=1}^{N}g_{ij}\right)/\left(2+2N\right)$ is the estimate of underlying allele frequency of SNP *j* (11). Compute *N*×*N* covariance matrix **Ψ**. Principal component analysis on ***X*** for subjects of both the training and testing data is used to obtain the eigenvectors, denoted as ***v***^*r*^, *r* = 1,⋯,*N*, and **Ψ*v***_*r*_ = *λ*_*r*_***v***_*r*_. Each vector *v*^*r*^∈ℝ^*N*^ corresponds to the *r*^*th*^ largest eigenvalue *λ*_*r*_. $v_{i}^{r}$ is the *i*^*th*^ loading of the eigenvector and carries the interpretation of *i*^*th*^ subject’s variation along the *r*^*th*^ ancestry axis. Suppose the top *q* eigenvectors contain a good amount of variation; $a_{r}=\lambda_{r}/\sum_{r=1}^{q}\lambda_{r}$ is the normalized eigenvalues. Compute the weighted score, $w=\sum_{r=1}^{q}a_{r}v^{r}\in R^{N}$, of which component *w*_*i*_ indicates the *i*^*th*^ subject’s variation summarized in the top *q* eigenvectors’ (ancestral) directions. By dividing ***w*** into *K* quantiles, the training subjects can be assigned to the *K* strata according to their quantile location in ***w***. The eigenvectors were obtained using the PLINK [14]. The *K* and *q* can be determined from cross-validations excluding the independent test data (**S1 Appendix**).

The probability of observing a subject belonging to a particular stratum can be approximated based on the distance of a subject to the stratum center. The center of stratum *k* is $c_{k}=\frac{1}{N_{k}}\sum_{i\in k}w_{i}$, where *N*_*k*_ is the number of subjects in the stratum. Position of a new subject *i* in the ancestral space can be calculated by $w_{i}=\sum_{r=1}^{q}a_{r}v_{i}^{r}$. As the squared distance of a subject to a cluster center empirically follows a chi-squared distribution, the probability that subject *i* belongs to stratum *k* can be estimated by,

$$\mathrm{Pr}\left(x_{i}|i\in k\right)=\mathrm{Pr}\left[\chi_{1}^{2}>{\left({\hat{\sigma }}_{i}^{2}\right)}^{-1}{\left(w_{i}-c_{k}\right)}^{2}\right],$$ (3)

where ${\hat{\sigma }}_{k}^{2}$ is the sample variance of *w*_*i*_ in the *k*^*th*^ stratum, *i*∈*k*.

In sum, the procedure of applying the PV is as follows: (1) Obtain eigenvectors and eigenvalues of all subjects genotypes (training and testing data) calculated in the ancestral direction; (2) divide the training set into *K* strata; (3) obtain stratum-wise predictors by a base prediction model in the training data, resulting *K* sets of predicted *Y* for the test data; (4) calculate the propensity of a test subject *i* to stratum *k* using **Eq 3**; (5) obtain the final predicted *Y* for the test set by **Eq 1**.

## Verification and comparison

### Simulation study I: applying PV in mixed population data

Simulation study I aims to investigate the effect of incorporating PV with an LMM base prediction model in dataset consists of multiple populations. The genotype data was obtained from two real GWAS of African and European populations from the GAIN project (dbGaP accession number: phs000021.v3.p2). We extracted data including 1,932 subjects of African ancestry (AA), 2,657 subjects of European ancestry (EA), and 9,242 common (MAF>1%) genetic variants of chromosome 22. The admixed population genotype data of 2,000 subjects was simulated by sampling a genetic variant *x*_*i*,*j*_ for subject *i* at locus *j* from a binomial distribution, $x_{i,j}\sim Bin(2,p_{j}^{E}Q_{i}^{E}+p_{j}^{A}Q_{i}^{A})$, where $p_{j}^{E}$ and $p_{j}^{A}$ are MAF of locus *j* in the EA and AA data, respectively; $Q_{i}^{E}$ and $Q_{i}^{A}$ are the ancestry fractions of subject *i* in the two populations; $Q_{i}^{E}+Q_{i}^{A}=1$ (**S1 Fig**). Three thousand causal variants were selected for the AA and EA population, respectively, among which 75% was common to both populations and 25% was unique to a single population [15]. Effect size was sampled from normal distributions with the number of variants in each effect group proportional to effect magnitude. Specifically, phenotype was determined by 10 SNPs of large effect from distribution *β*~N(0, 10^−2^), 300 SNPs of moderate effects with *β*~N(0, 10^−3^), and the remaining variants from *β*~N(0, 10^−4^) [16]. Risk effect of the admixed population was simulated by setting $\beta_{j}^{i}=Q_{i}^{E}\beta_{j}^{E}+Q_{i}^{A}\beta_{j}^{A},j=1,2,\ldots ,3000$, where $\beta_{j}^{E}$ and $\beta_{j}^{A}$ represent effect sizes of SNP *j* in EA and AA, respectively. A linear model was used to obtain phenotype of subjects from the causal variants and effect sizes, in which the residual term follows a normal distribution of variance satisfying alternative heritability scenarios (*h*^2^ = 0.2, 0.5 and 0.8). In the mixed data consisted of the EA, AA, and admixed populations, PV was implemented with base prediction models controlling for the top ten PCs, and by the reference methods that are the base models controlling for PCs only. For the base models, we considered the linear regression model (LM), BayesR [16], and Dirichlet Process Regression (DPR) [17]. The BayesR is a linear mixed model (LMM) assuming the effect of variants follows a normal mixture distribution with the majority of variants having no effect on the phenotype. While the other LMM method, DPR, adopts a non-parametric prior on effect distributions and assigns non-zero effect on all variants. True ancestry information of subjects was treated as unknown and was controlled purely through statistical modelling. Selection for *K* and *q* can be found in **S1 Appendix**. Throughout the simulation and real data application in this study, prediction accuracy is measured by Pearson correlation coefficient between the observed and predicted outcome for continuous phenotypes, and by area-under-the-curve (AUC) for binary outcomes. Averaged prediction accuracy on independent test sets in the five-group cross-validation (5GCV) was reported.

**Fig 2:**
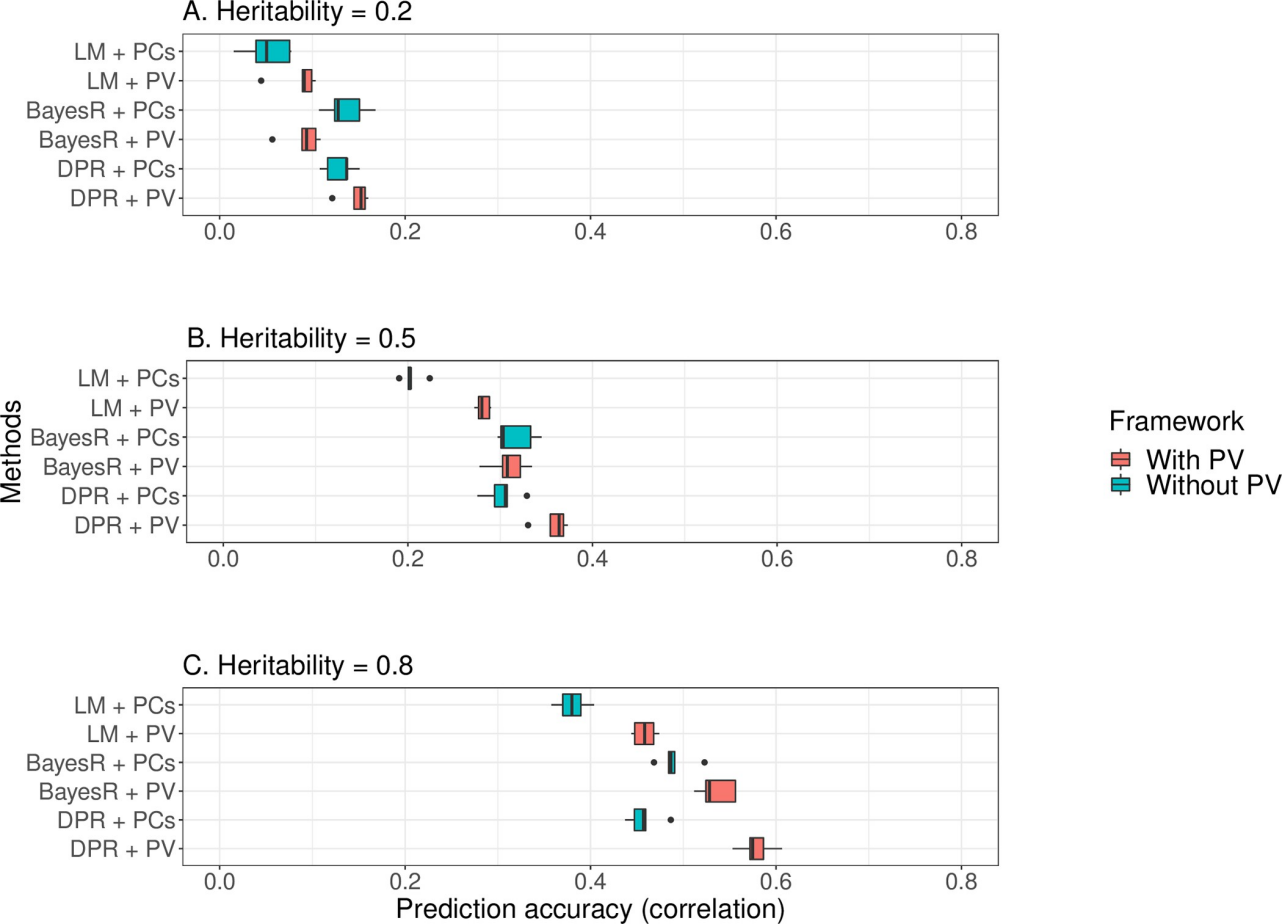
Prediction outcome of the Prism Vote implemented with alternative base prediction models (Simulation Study I). **Legend**: With PV: Prediction model is the Prism Vote with the DPR base controlling top 10 PCs; Without PV (the reference method): DPR controlling for the top 10 PCs only. Panels **(A)** Heritability = 0.2; **(B)** Heritability = 0.5; and **(C)** Heritability = 0.8. Base models include the linear regression model (LM), BayesR, and Dirichlet process regression (DPR). For all base models, the PV generally improves mean prediction accuracy in terms of concordance correlation coefficient in 5GCV compared to the reference methods. Detailed results can be found in **S2 Appendix**.

**Fig 2** showed that the PV generally improved prediction accuracy of the base models comparing to the reference methods in 5GCV. Under the high heritability scenario, the PV improved the mean prediction correlation coefficient of the BayesR from 0.49 to 0.54 by 10.2%, and improved the DPR from 0.46 to 0.58 by 26.5% (**Fig 2**, **S2 Appendix. Table**). PV improved the LM and DPR in all heritability scenarios, while it only enhanced BayesR under the high heritability setting. This might be due to the sparse effect model assumption made by the BayesR, from which modest causal effects were prevailingly estimated as zero in genetic data of low heritability.

### Simulation study II: Prediction performance as genetic heterogeneity varies

**Fig 3:**
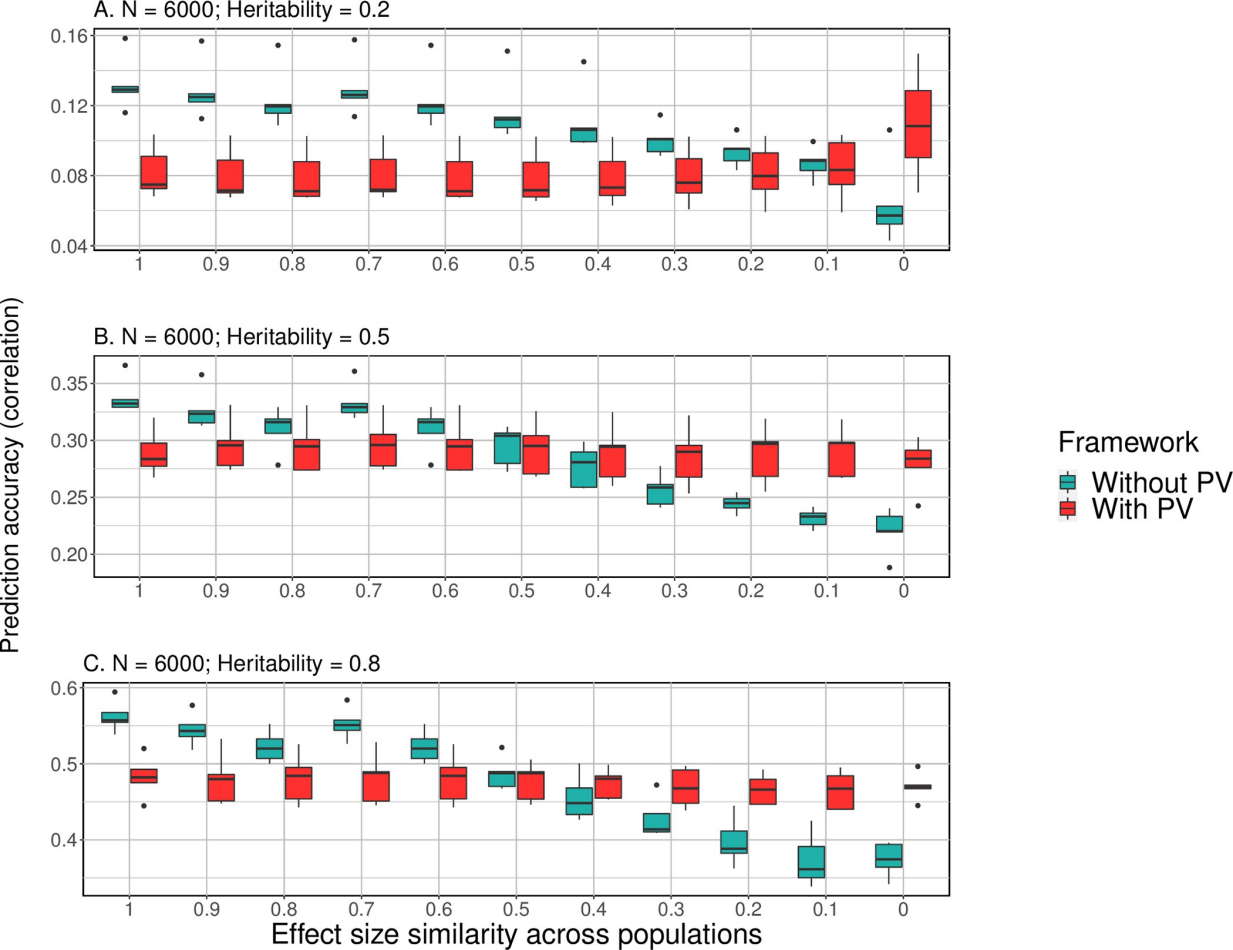
Prediction performance of PV as genetic heterogeneity increases across populations (Simulation Study II). **Legend**: With PV: Prediction model is the Prism Vote with the DPR base controlling top 10 PCs; Without PV (the reference method): DPR controlling for the top 10 PCs only. **(A)** Heritability = 0.2; **(B)** Heritability = 0.5; **(C)** Heritability = 0.8. Vertical axis: average correlation coefficient of the predicted and observed phenotype in 5GCVs. Horizontal axis: levels of effect size similarity across populations. As effect size similarity decreased across populations, the PV shows stable prediction outcomes (red), while performances of the reference method were affected substantially (green).

In simulation study II, we investigate the performance of PV with DPR base as genetic heterogeneity across populations varies. Genotype data was generated according to the minor allele frequency distribution from the EA and AA populations. Two thousand subjects were simulated for each of the single and admixed populations. Genetic similarity was controlled by covariance of effect size in the EA and AA populations. Let ***β***^*k*^∈ℝ^*m*^ denote effect of *m* causal SNPs in population *k*, which follows a multivariate normal distribution [18],

$$\left[\begin{array}{c} \beta^{k}\\ \beta^{k^{\prime }} \end{array}\right]∼N(0,[\begin{array}{cc} \frac{\sigma_{k}^{2}}{m}I_{m} & \frac{\rho_{k,k\prime }}{m}I_{m}\\ \frac{\rho_{k,k\prime }}{m}I_{m} & \frac{\sigma_{k^{\prime }}^{2}}{m}I_{m} \end{array}]),$$

in which $\sigma_{k}^{2}$ and $\sigma_{k^{\prime }}^{2}$ are variance of the total additive genetic effect of these SNPs in two populations *k* and *k*′, *k*≠*k*′∈{1,⋯,*K*}. The covariance *ρ*_*k*,*k*′_ approximates the “shared heritability”. Thus, genetic similarity of two populations can be measured by the ratio *η* = *ρ*_*k*,*k*′_/(*σ*_*k*_*σ*_*k*′_). When *η* = 0, the populations share no effect similarity; and as *η* approaches one, the traits are influenced by similar genetic effects in the mixed populations. As shown in **Fig 3**, the reference group (DPR + PCs)’s prediction accuracy observes substantial reduction as genetic similarity in populations decreases, while DPR implemented in the PV framework produces stable prediction accuracies in all scenarios. For instance, under the high heritability scenario (**Fig 3C**), as effect similarity decreases from 80% to 20%, mean prediction accuracy by the base model in the reference group reduces from 0.52 to 0.40 by 23.1%, while the accuracy with PV only slightly drops from 0.48 to 0.47 by 2.1%. Under the medium and high heritability settings (**Fig 3B** and **3C**), prediction gain by the PV is warranted when the genetic similarity in multiple populations is lower than half.

### Simulation study III: Prediction performance as sample size increases

**Fig 4:**
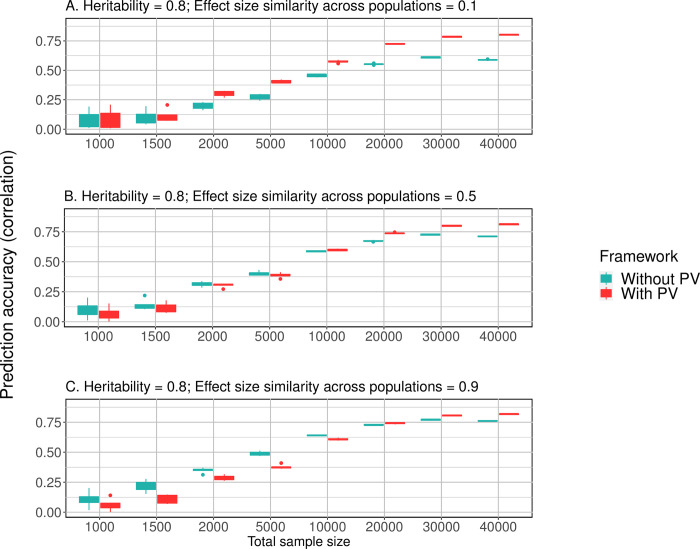
Prediction performance of PV as sample size increases (Simulation study III). Legend: With PV: Prediction model is the Prism Vote with the DPR base controlling top 10 PCs; Without PV (the reference method): DPR controlling for the top 10 PCs only. **(A)** Heritability = 0.8, effect size similarity *η* = 0.1; **(B)** Heritability = 0.8, *η* = 0.5; **(C)** Heritability = 0.8, *η* = 0.9. As the sample size increased from 1,000 to 40,000, the prediction accuracy of PV continued to increase. The PV’s advantage was more evident when genetic heterogeneity was high (Panel A and B). Results for scenarios of heritability = 0.2 and 0.5 can be found in **S3 Appendix**.

This simulation considers influence of sample size on prediction accuracy. In each combination of heritability (*h*^2^ = 0.2, 0.5 and 0.8) and genetic similarity (*η* = 0.1, 0.5, 0.9) category, eight datasets were simulated at different sample sizes (*N* = 1,000 to 40,000). As sample size of data steadily increases, implementing PV results in prediction accuracy gain in all nine scenarios (**Fig 4** and **S3 Appendix**). Particularly, when *N* = 40,000, *h*^2^ = 0.8 and *η* = 0.1, PV improved the prediction accuracy of DPR from 0.59 to 0.80 by 26.3% (**Fig 4A**).

## Applications

The first real GWAS data for application is from the Population Architecture through Genomics and Environment (PAGE) project (dbGap accession number phs000220.v2.p2). A total number of 9,075 subjects were extracted, consisted of 3,520 self-identified African, 2,104 Hawaiians, and 3,451 Japanese (**S4 Appendix. Fig A**). Quality control (QC) was performed by removing SNPs with genotype call rate < 95%, Hardy-Weinberg equilibrium (HWE) *p*-value < 5×10^−8^ or MAF < 0.01. After QC, 560,899 autosomal SNPs were available for analysis. The second GWAS is a subset of the non-European population in the UK Biobank [19]. We included 5,718 individuals of Indian ancestry, 4,297 Caribbean, 3,204 African, 1,748 Pakistan 1,504 Chinese, 221 Bangladeshi, 2,869 admixed populations, and 6,947 subjects without clear ancestry information (**S4 Appendix. Fig B**). After QC, 26,506 subjects and 524,557 SNPs were available for analysis. In the PAGE data, traits including the body mass index (BMI), height, diabetes, and hypertension were analyzed; and in the UK biobank data, BMI, height, cardiovascular disease (CVD), and diabetes diagnosed by doctor (diabetes) were analyzed. For both datasets, the optimal stratum number was estimated to be two, and *q* was set to ten.

**Fig 5:**
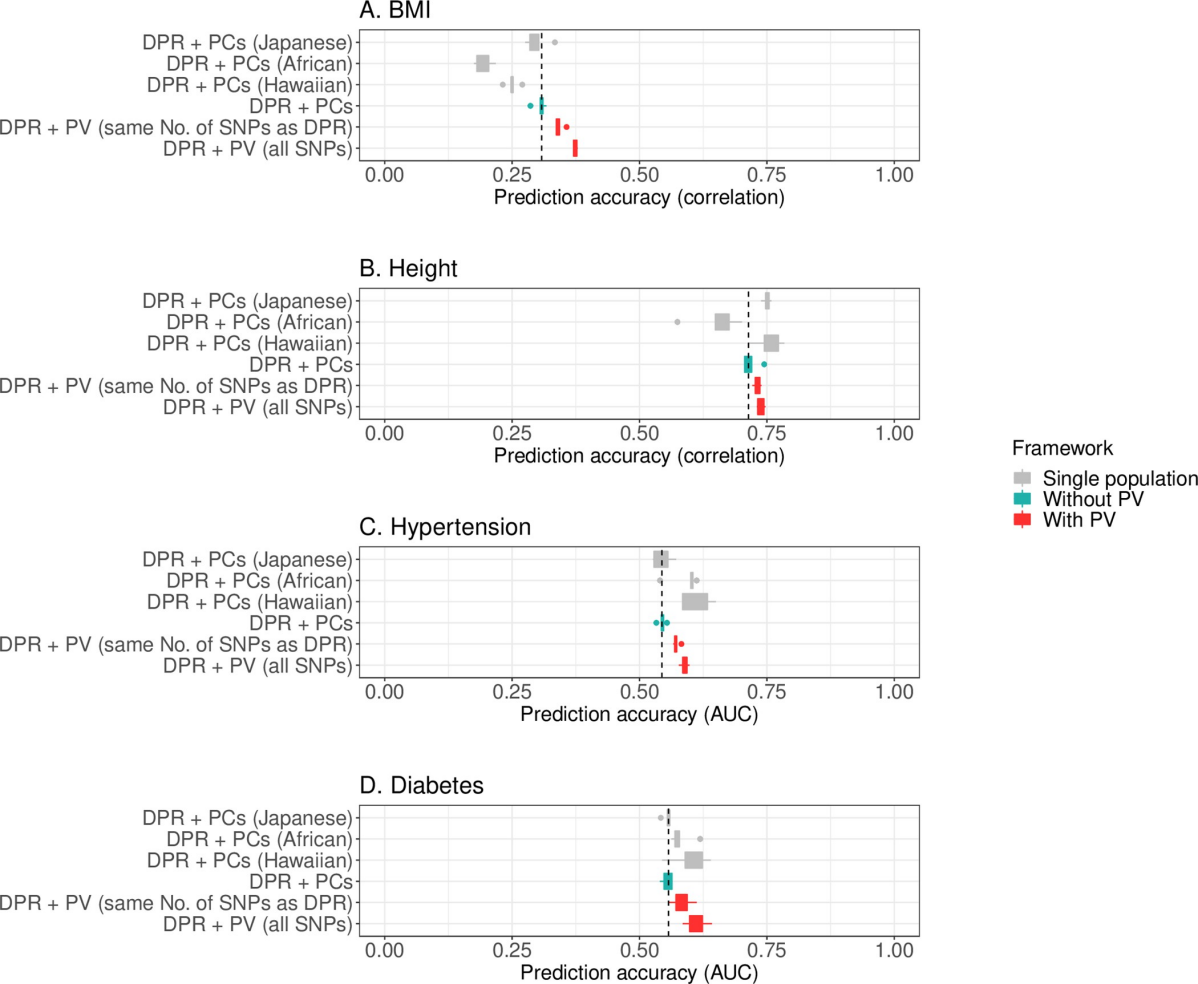
Prediction performance of PV in real data application (PAGE dataset). **Legend**: Mean 5GCV Prediction accuracy of PV and the reference method using DPR base model in mixed populations of the PAGE data. Comparing to the reference method, PV enhances the prediction accuracy of DPR by 12.1% (SD 4.7%) for the BMI, 2.0% (1.5%) for height, 5.2% (SD 2.1%) for hypertension, and 5.4% (SD 2.2%) for diabetes. Prediction outcome in single populations by the reference method is shown in gray color. Details can be found in **S1 Table**.

In the PAGE data, PV was implemented with DPR controlling PCs for predicting BMI, height, diabetes, and hypertension (**Fig 5**). Comparing to the reference method (DPR+PCs), PV enhanced the prediction accuracy of base model by 12.1% (SD 4.7%) for BMI, 2.0% (1.5%) for height, 5.2% (SD 2.1%) for hypertension, and 5.4% (SD 2.2%) for diabetes. For easier interpretation of the results, we also displayed the prediction outcome achieved in single populations (**Fig 5, S1 Table**). In general, by the reference method, prediction accuracy in the joint cohort is in between the highest and lowest performance achieved in single populations (**Fig 5B–5D**), while the PV elevates the prediction outcome in mixed population data close to the best accuracy reached in single populations. For example, in **Fig 5D**, the prediction for diabetes by the reference method is in-between its performance in the Japanese population that is observed with the lowest accuracy and the African population the second lowest; while the PV improves the joint cohort prediction to an accuracy achieved in the Hawaiian population with the highest performance. Furthermore, as shown in **Fig 5A**, PV increases prediction accuracy for BMI to 0.374 (SD 0.004) in the mixed population, a level unreached in single populations, among which the best performance is only 0.299 (SD 0.022).

**Fig 6:**
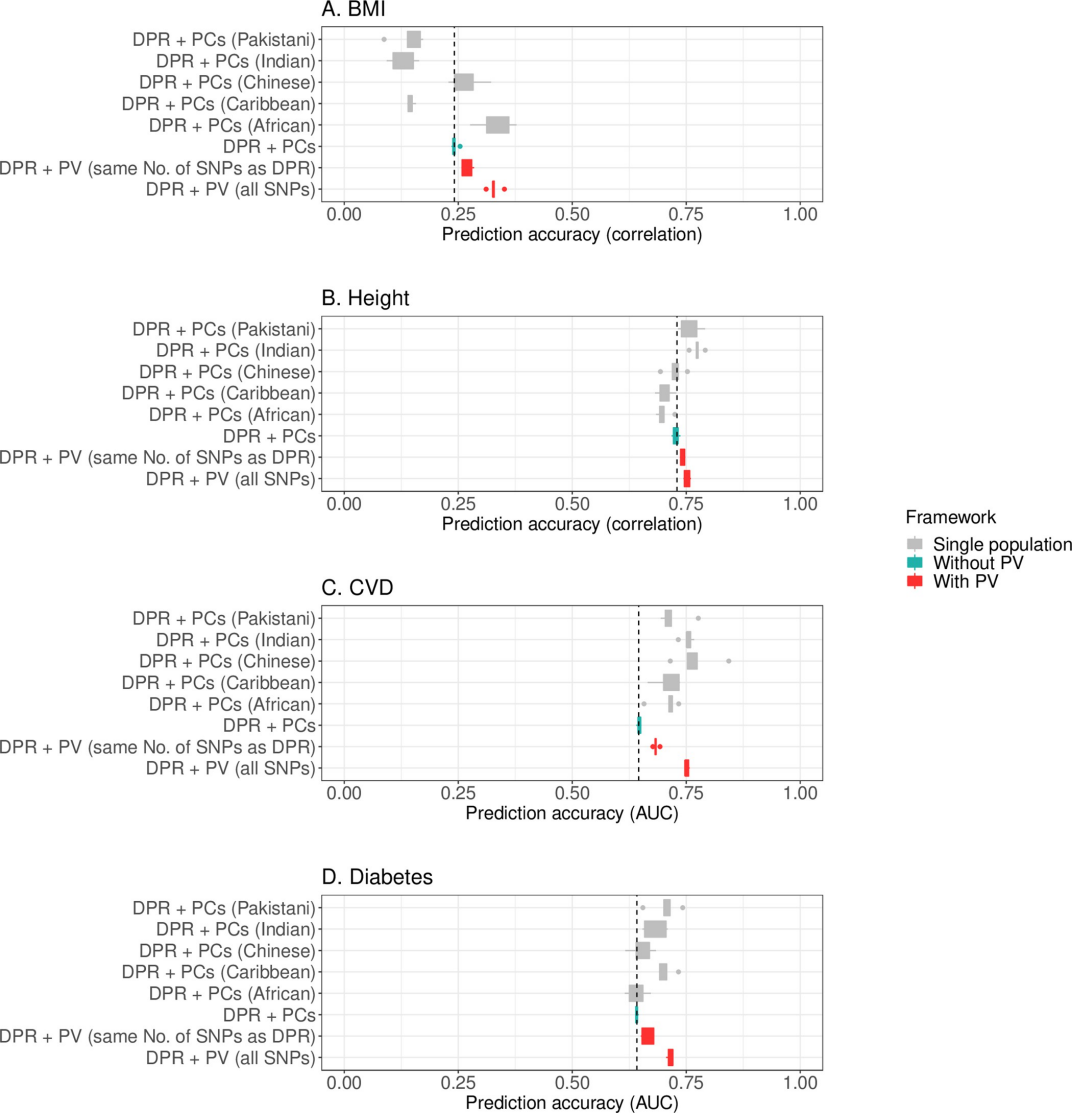
Prediction performance of the PV in real data application (UK Biobank data). **Legend**: Mean 5GCV prediction accuracy of the PV and reference method using the DPR base model in the UK Biobank mixed population (non-European) data. Applying the PV significantly improved the prediction accuracy of DPR for BMI by 12.0% (SD 5.6%), for height by 1.44% (SD 0.38%), for CVD by 5.9% (SD 1.0%), and for diabetes by 3.7% (SD 2.2%). Details can be found in **S3 Table**.

In UK Biobank data composed of five minority populations and subjects of 12 vague self-reported ancestries (**S2 Table**), applying PV with the DPR base significantly improves the prediction accuracy for the BMI by 12.0% (SD 5.6%), for the height by 1.44% (SD 0.38%), for the CVD by 5.9% (SD 1.0%), and for the diabetes by 3.7% (SD 2.2%) (**Fig 6, S3 Table**). Prediction standard deviations also considerably reduce in the joint data analysis, benefited from the larger sample size. Finally, we compared the estimated effect size of the top 5,000 SNPs in the two real GWAS datasets (**S5 Appendix. Figs A and B**); the effect sizes are vastly different between stratum, suggesting prevailing genetic heterogeneity in these data.

## Discussion

The Prism Vote framework is introduced to dissect and integrate risk of individuals based on personalized risk spectrum through a Bayesian probability framework. Simulation studies showed that the method generally enhanced the prediction of base models in different heritability scenarios; and advantage of the framework expanded with increasing genetic heterogeneity and sample size. Application of the PV in two real GWASs data of mixed populations also resulted considerable gain in prediction accuracy.

The component steps of the framework can be substituted with alternative methods according to data attributes. In the population stratification step, either a model-based or model-free model may be incorporated [20–22]. The two approaches were tested on a simulated admixed cohort generated from two distinct populations (**S6 Appendix**). Individuals’ group membership probabilities obtained by the PC-based method described in this study gave concordant estimates as the outcome obtained from the Bayesian maximum likelihood approach implemented in the ADMIXTURE software [22] (**S6 Appendix**).

For the prediction step, linear mixed model is pertinent for this study design for its good property of simultaneous estimation of whole genome SNPs effects and prediction. Other approaches, such as the machine learning methods, or the PRS, may be applied to construct predictors in stratum. To apply the PRS, the following issues shall be considered. The PRS draws summary statistics from well-powered external datasets, however, these external populations were predominantly of the European ancestries. Therefore, although coefficient of the aggregated PRS can be evaluated in stratum, to differentiate SNP-effects across stratum, one need to estimate the transferrable part of the effect size from auxiliary data to strata [10,23], or to construct joint PRS for the mixed populations in strata [24]. These would rely on the feasibility of calculating the transferrable genetic effect or the availability of ancestry-specific summary statistics. We explored implementation of PV with the joint PRS approach in the PAGE data. On the BMI trait, using LDpred2 [25] as base model in mixed populations [24] (**S7 Appendix**), PV significantly improved the prediction correlation coefficient from 0.372 (SD 0.013) to 0.389 (SD 0.015) in 5GCV comparing to using the base model with PCs. Nevertheless, no significant difference was observed in the prediction for the other traits (**S7 Appendix. Table B**).

The PV framework leverages on data’s genetic architecture to form homogeneous genetic strata. The grouping of subjects is a complicated issue as it is simultaneously influenced by the sample size, underlying genetic models of the trait, and genetic architecture in strata. In either model-based or model-free approach, the number of population clusters was often determined empirically [5,20,21]. In the current analysis, an equal division approach was adopted such that each stratum has the same sample size. Nevertheless, the clustering step could be further optimized by considering bias-variance trade-off for SNP-effects estimation within stratum towards achieving optimal prediction outcome, which requires extensive research in future studies.

One notable advantage of the PV framework is that it enables prediction for subjects with unknown or admixed ancestries by decomposing subject’s propensity to more homogeneous subpopulation stratum, thereby allowing the extraction of information from other populations to inform prediction of admixed samples (**S8 Appendix**). Another advantage of the PV is that it allows distributed programing of large genomic datasets in the dimension of subjects. Traditionally, SNPs are assigned to multiple clusters to increase computation efficiency, yet distributed computing is difficult to be carried out for the prediction models requiring simultaneous evaluation of biomarkers, such as the LMM or penalized regression. Markedly, the PV framework enables the simultaneous evaluation and prediction incorporating all SNPs in distributed calculations, by applying the prism filter on individuals and estimating disease risk from genetic background of the subpopulations that are assigned to CPU-clusters. Meanwhile, the PV’s Bayesian probability framework maintains total information gain from the subpopulations, producing a balanced and potentially improved prediction outcome.

In this study, we proposed the Prism Vote method for predicting human complex traits in genotype data consisted of multiple populations, and investigated application of the prism filter in the aspect of genetic similarity of subjects. The framework might be extended to alternative stratification aspects such as phenotype subgroups for improving prediction of a particular trait, as well as to other genetic or non-genetic datasets, which will be explored in future studies.

## Supporting information

S1 Appendix Selecting the optimal K and q. S1 Appendix. Fig A. Selecting optimal K and q in simulation data I when true K = 1. S1 Appendix. Fig B. Selecting optimal K and q in simulation data I when true K = 2. S1 Appendix. Fig C. Selecting optimal K and q in simulation data I when true K = 3.
(DOCX)

S2 Appendix Additional results of Simulation Study I. S2 Appendix. Table. The Pearson correlation of predicted phenotype and true phenotype using different methods (Simulation Study I). S2 Appendix. Fig. Compare prediction accuracy in single and mixed populations (Simulation study I).
(DOCX)

S3 Appendix Additional results of Simulation Study III. S3 Appendix. Fig A. Prediction performance of PV with increasing sample size, heritability = 0.2 (Simulation Study III). S3 Appendix. Fig B. Prediction performance of PV with increasing sample size, heritability = 0.5 (Simulation Study III).
(DOCX)

S4 Appendix The genetic ancestries in real data applications. S4 Appendix. Fig A. PAGE data. S4 Appendix. Fig B. The genetic ancestry of minority populations in UK Biobank.
(DOCX)

S5 Appendix Effect size stratification by subpopulation. S5 Appendix. Fig A. Effect size stratification by subpopulations—PAGE data. S5 Appendix. Fig B. Effect size stratification by subpopulations—UK Biobank data.
(DOCX)

S6 Appendix The concordance of PV probability with ADMIXTURE (Simulation Study IV). S6 Appendix. Fig A. genetic ancestries in simulation study IV. S6 Appendix. Fig B. Comparing genetic ancestry fraction estimated by PV and ADMIXTURE.
(DOCX)

S7 Appendix Implementing PV with summary statistics in mixed populations. S7 Appendix. Fig A. PCA projections of the subjects from UK Biobank colored by inferred ancestry. S7 Appendix. Table A. Sample size of inferred ancestry populations in the UK Biobank data. S7 Appendix. Table B. Implementation of PV using LDpred2 as base model in the PAGE data.
(DOCX)

S8 Appendix Prediction accuracies in various train-test population combinations. S8 Appendix. Table A. PAGE data. S8 Appendix. Table B. UK Biobank data.
(DOCX)

S1 Fig The genetic ancestries in simulation study I. (DOCX)

S1 Table Prediction performance of the PV in real data application (PAGE dataset). (DOCX)

S2 Table Sample size of non-European populations in the UK Biobank. (DOCX)

S3 Table Prediction performance of the PV in real data application (UK Biobank). (DOCX)
