# Supplementary material for: A Prism Vote method for individualized risk prediction of traits in genotype data of Multi-population
Source: PLoS Genet. 2022 Oct 27;18(10):e1010443. doi: 10.1371/journal.pgen.1010443 (PMC9642904; doi:10.1371/journal.pgen.1010443)
Supplement: S1 Appendix — Fig A. Selecting optimal K and q in simulation data I when true K = 1. S1 Appendix. Fig B. Selecting optimal K and q in simulation data I when true K = 2. S1 Appendix. Fig C. Selecting optimal K and q in simulation data I when true K = 3. (DOCX) [file pgen.1010443.s001.docx]

# S1 Appendix: Selecting the optimal *K* and *q*

The two parameters (*K* and *q*) can be determined via adding a cross-validation layer apart from the independent test set. Partition the data into train, validation, and test set in ratios of 7:1:2. Tuning parameters are estimated by maximizing the prediction accuracy on the validation sets. The final prediction performance is evaluated by the averaged prediction accuracy on the independent test sets in 5-group cross-validation (5GCV).

***Simulation study for selecting tuning parameters***

Real genotype data is extracted from three single populations of UK Biobank. Phenotype of subjects was simulated in a similar way to Simulation I (**Materials and Methods**). By setting true *K* = 1, 2 and 3, we calculated average prediction accuracy using DPR+PV on the *validation* group (**S1-Figure A, B and C)**. In all scenarios, the validation groups select the correct number of stratums. Varying the number of eigenvectors *q* between 5 to 50 show that the prediction accuracy is robust. A default value of *q* = 10 can be adopted.

## S1 Appendix. Fig A. Selecting optimal *K* and *q* in simulation data I when true *K*=1


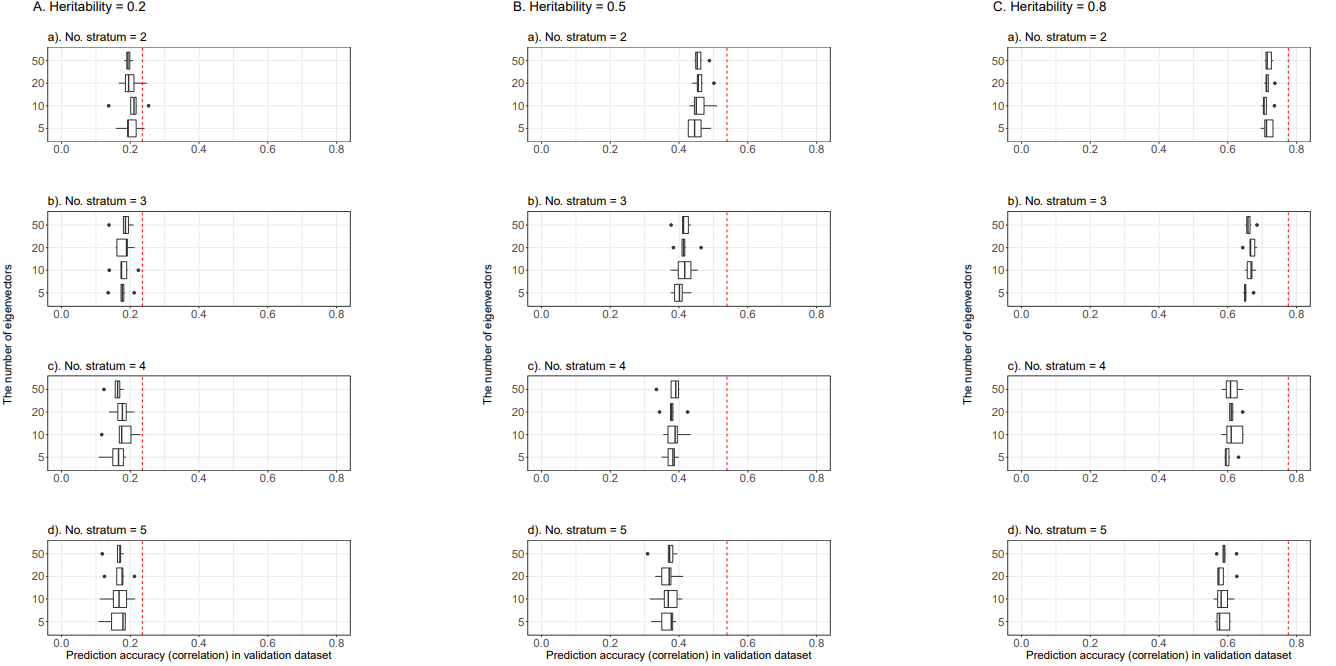


**Legend**: The red dashed line indicates the prediction accuracy at *K*=1 (without stratification) calculated using the reference method (DPR+PCs). As true *K* =1, the validation results correctly suggest that the data shall not be divided.

## S1 Appendix. Fig B. Selecting optimal *K* and *q* in simulation data I when true *K*=2

**Legend**: The red dashed line indicates the prediction accuracy at *K*=1 (without stratification) calculated using the reference method (DPR+PCs). When true *K* =2, the validation group correctly indicates that two stratum shall be adopted in PV.

## S1 Appendix. Fig C. Selecting optimal *K* and *q* in simulation data I when true *K*=3

**
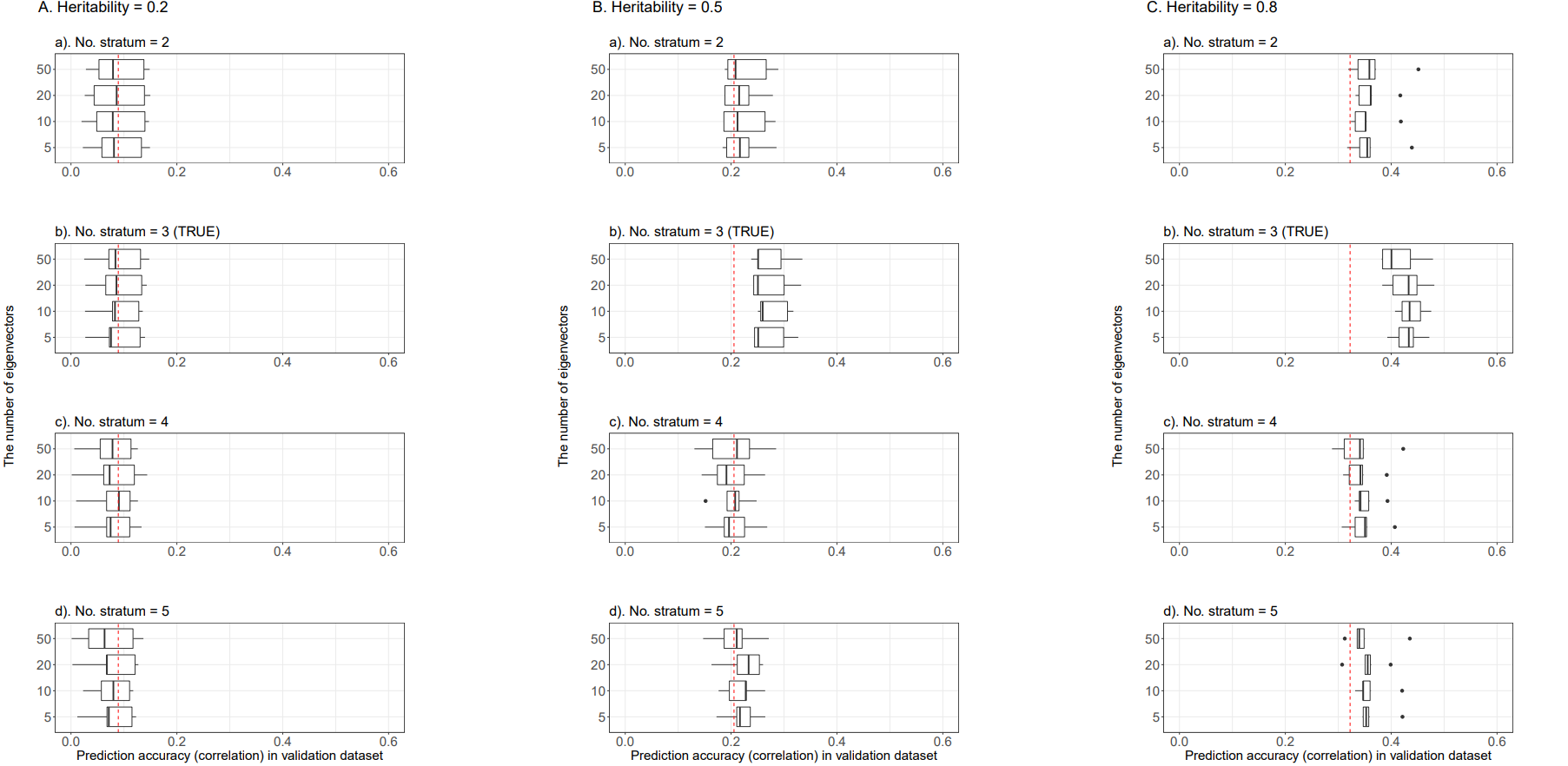
**

**Legend**: The red dashed line indicates the prediction accuracy at *K*=1 (no stratification) obtained by DPR+PCs. Cross-validation layer infers that three stratum shall be adopted.
