# Supplementary material for: A Prism Vote method for individualized risk prediction of traits in genotype data of Multi-population
Source: PLoS Genet. 2022 Oct 27;18(10):e1010443. doi: 10.1371/journal.pgen.1010443 (PMC9642904; doi:10.1371/journal.pgen.1010443)
Supplement: S2 Appendix — Table. The Pearson correlation of predicted phenotype and true phenotype using different methods (Simulation Study I). S2 Appendix. Fig. Compare prediction accuracy in single and mixed populations (Simulation study I). (DOCX) [file pgen.1010443.s002.docx]

# S2 Appendix. Additional results of Simulation Study I

## S2 Appendix. Table: The Pearson correlation of predicted phenotype and true phenotype using different methods (Simulation Study I).

| Averaged correlation (SD) by models: |  | Heritability |  |
| --- | --- | --- | --- |
|  | 0.2 | 0.5 | 0.8 |
| LM + PCs | 0.052 (0.026) | 0.204 (0.012) | 0.380 (0.018) |
| LM + PV | 0.086 (0.024) | 0.281 (0.008) | 0.458 (0.013) |
| BayesR + PCs | 0.136 (0.024) | 0.316 (0.022) | 0.491 (0.020) |
| BayesR + PV | 0.090 (0.020) | 0.309 (0.021) | 0.535 (0.020) |
| DPR + PCs | 0.130 (0.017) | 0.302 (0.020) | 0.458 (0.018) |
| DPR + PV | 0.147 (0.015) | 0.358 (0.017) | 0.579 (0.020) |

## S2 Appendix. Fig: Compare prediction accuracy in single and mixed populations (Simulation study I)

**Legend:** Parenthesis indicates the single population where the methods are applied. EA: European Ancestry, AA: African Ancestry, Admixed: admixed population. “PDR+PCs” and “DPR+PV” rows indicate the prediction accuracy obtained in mixed populations.
