# Supplementary material for: A Prism Vote method for individualized risk prediction of traits in genotype data of Multi-population
Source: PLoS Genet. 2022 Oct 27;18(10):e1010443. doi: 10.1371/journal.pgen.1010443 (PMC9642904; doi:10.1371/journal.pgen.1010443)
Supplement: S3 Appendix — Fig A. Prediction performance of PV with increasing sample size, heritability = 0.2 (Simulation Study III). S3 Appendix. Fig B. Prediction performance of PV with increasing sample size, heritability = 0.5 (Simulation Study III). (DOCX) [file pgen.1010443.s003.docx]

# S3 Appendix. Additional results of Simulation Study III

## S3 Appendix. Fig A. Prediction performance of PV with increasing sample size, heritability = 0.2 (Simulation Study III)

**Legend:** At heritability level 0.2, comparing performance of prediction framework with PV and without PV (**Materials and Methods**). The advantage of PV is more evident when the effect size similarity across populations is low (Panel A), than in the scenario when effect size similarity is high (Panel C). As sample size increases, PV eventually outperforms the model without PV in all scenarios.

## S3 Appendix. Fig B. Prediction performance of PV with increasing sample size, heritability= 0.5 (Simulation Study III)

**Legend:** At heritability level 0.5, comparing performance of prediction framework with PV and without PV (**Materials and Methods**). The advantage of PV is more evident when the effect size similarity across populations is low (Panel A), than in the scenario when effect size similarity is high (Panel C). As the sample size increases, PV eventually outperforms the model without PV in all scenarios.
