# Supplementary material for: A Prism Vote method for individualized risk prediction of traits in genotype data of Multi-population
Source: PLoS Genet. 2022 Oct 27;18(10):e1010443. doi: 10.1371/journal.pgen.1010443 (PMC9642904; doi:10.1371/journal.pgen.1010443)
Supplement: S4 Appendix — Fig A. PAGE data. S4 Appendix. Fig B. The genetic ancestry of minority populations in UK Biobank. (DOCX) [file pgen.1010443.s004.docx]

# S4 Appendix. The genetic ancestries in real data applications

## S4 Appendix. Fig A. PAGE data

**
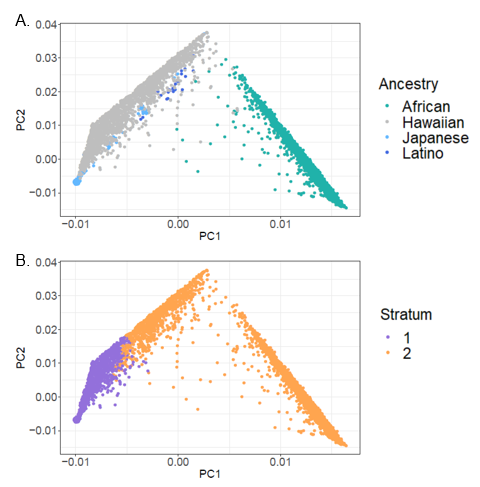
**

**Legend:** A. The populations plotted in the coordinates spanned by the top two principal components (PCs) annotated by ancestry information. B. The distribution of subjects in two strata when we stratify all subjects by the median of weighted sum of top 10 PCs.

## S4 Appendix. Fig B. The genetic ancestry of minority populations in UK Biobank.

**
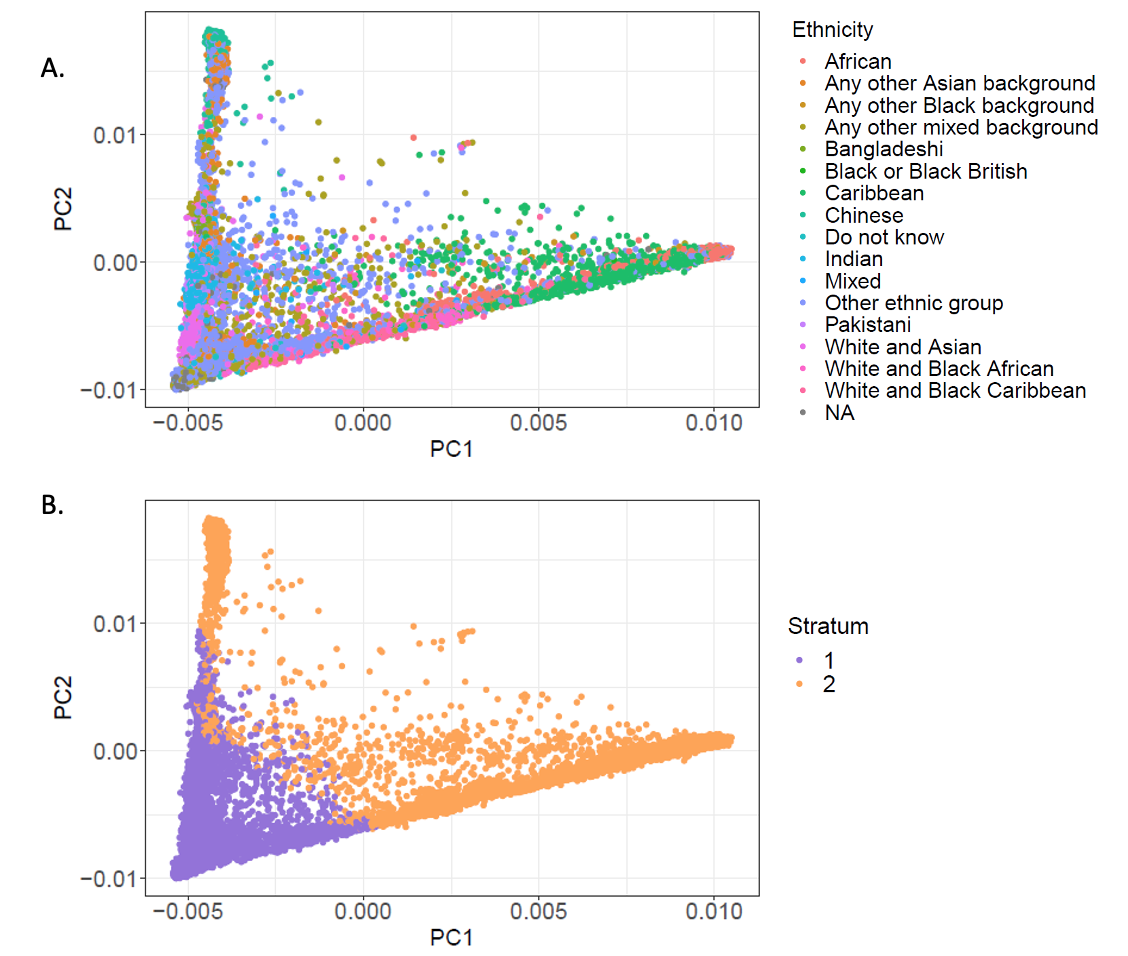
**

**Legend:** A. The populations plotted in the coordinates spanned by the top two principal components (PCs) annotated by ancestry information. B. The distribution of subjects in two strata when we stratify all subjects by the median of weighted sum of top 10 PCs.
