# Supplementary material for: A Prism Vote method for individualized risk prediction of traits in genotype data of Multi-population
Source: PLoS Genet. 2022 Oct 27;18(10):e1010443. doi: 10.1371/journal.pgen.1010443 (PMC9642904; doi:10.1371/journal.pgen.1010443)
Supplement: S5 Appendix — Fig A. Effect size stratification by subpopulations—PAGE data. S5 Appendix. Fig B. Effect size stratification by subpopulations—UK Biobank data. (DOCX) [file pgen.1010443.s005.docx]

# S5 Appendix. Effect size stratification by subpopulation

## S5 Appendix. Fig A. Effect size stratification by subpopulations - PAGE data

**
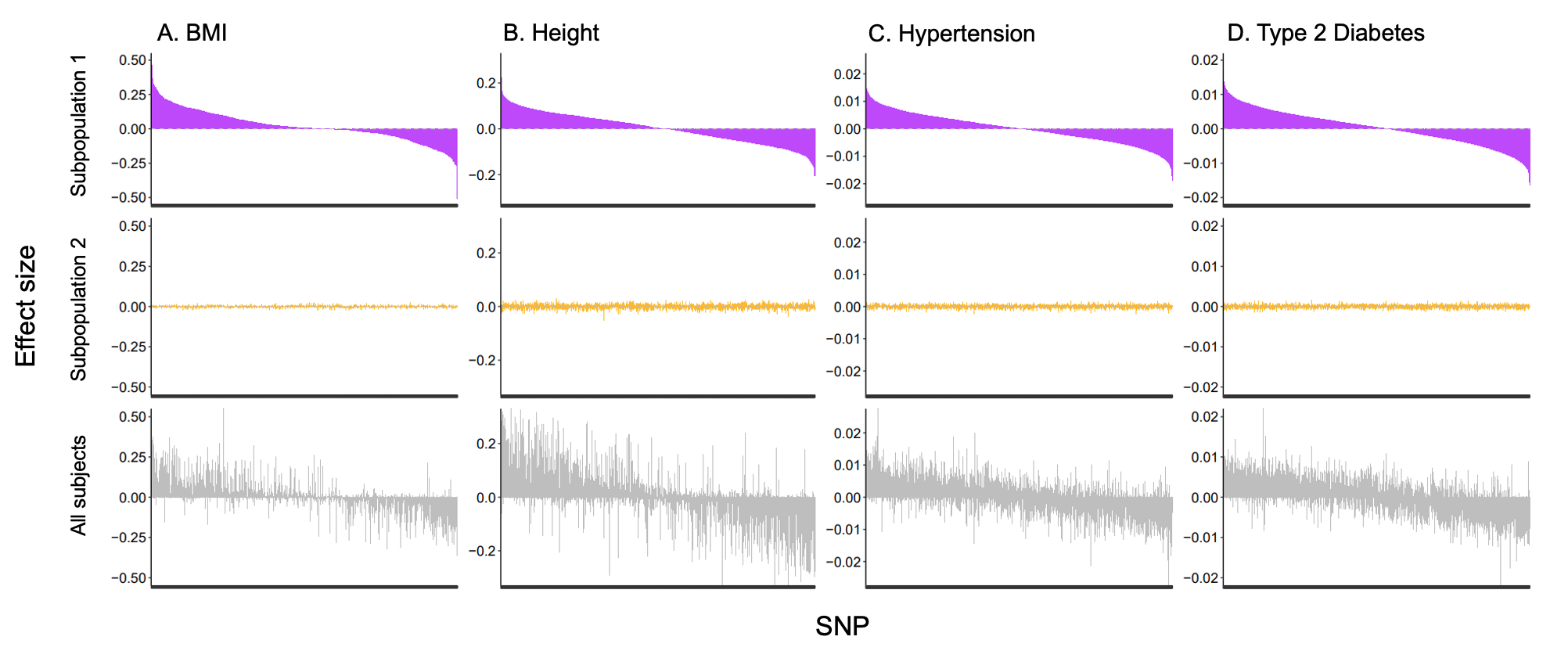
**

**Legend:** Top 5,000 significant SNPs are selected in subpopulation 1 and their effect sizes are estimated by the DPR in both subpopulations. Horizontal axis: SNPs ranked by effect size estimated in subpopulation 1.

## S5 Appendix. Fig B. Effect size stratification by subpopulations - UK Biobank data.

**
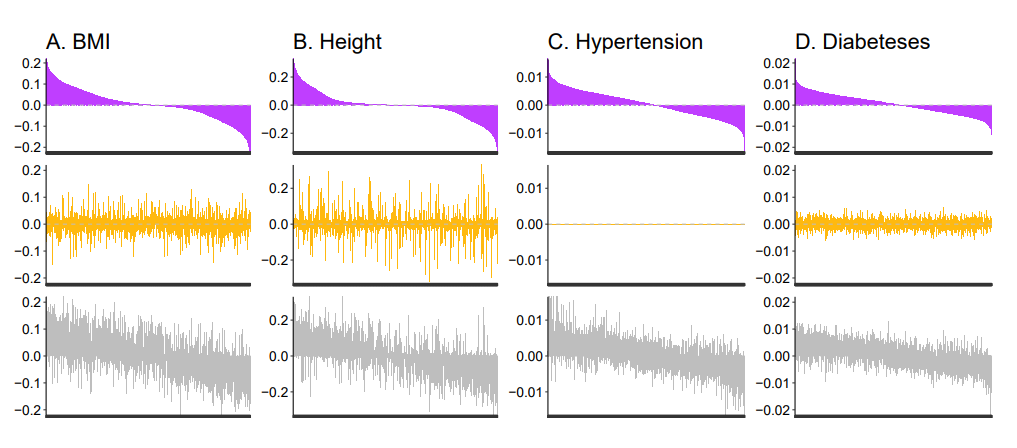
**

**Legend:** Top 5,000 significant SNPs are selected in subpopulation 1 and their effect sizes are estimated by the DPR in both subpopulations. Horizontal axis: SNPs ranked by effect size estimated in subpopulation 1. For CVD (panel C), the estimated effect sizes in subpopulation 2 is around the magnitude of ${10}^{-5}$ .
