# Supplementary material for: A Prism Vote method for individualized risk prediction of traits in genotype data of Multi-population
Source: PLoS Genet. 2022 Oct 27;18(10):e1010443. doi: 10.1371/journal.pgen.1010443 (PMC9642904; doi:10.1371/journal.pgen.1010443)
Supplement: S6 Appendix — Fig A. genetic ancestries in simulation study IV. S6 Appendix. Fig B. Comparing genetic ancestry fraction estimated by PV and ADMIXTURE. (DOCX) [file pgen.1010443.s006.docx]

# S6 Appendix. The concordance of PV probability with ADMIXTURE (Simulation Study IV)

In this simulation study, we assess the concordance of individual genetic ancestry estimated by the PV framework and the ADMIXTURE [1] method. Referencing real GWAS data of European and African populations (dbGaP accession number: phs000021.v3.p2), an admixture population and two non-admixed “pure” ancestry cohorts consisting of 6,000 subjects and 10,000 SNPs were simulated (**S6 Appendix. Fig A**). We calculated the PV probability of admixture population subjects assuming the two non-admixed cohorts are the two training strata. The calculated PV probability ${\Pr\left( \boldsymbol{x}_{s}|s\in k \right)}/{\sum_{k} \Pr\left( \boldsymbol{x}_{s}|s\in k \right)}$ matched closely to the genetic ancestry fraction estimated by ADMIXTURE with Pearson correlation = 96.44% (**S6 Appendix. Fig B**).

## S6 Appendix. Fig A. genetic ancestries in simulation study IV

**Legend: (A)** The inferred ancestries of the simulated admixture population; **(B)** The admixed population plotted in the coordinates spanned by the top two principal components (PCs).

## S6 Appendix. Fig B. Comparing genetic ancestry fraction estimated by PV and ADMIXTURE

**Legend**: PV and ADMIXTURE are applied to simulation data IV to calculate the genetic ancestry proportion of the the admixed samples to one of the single populations. In ADMIXTURE, *K* = 2; and in PV, *K* = 2, *q*=10.

# References

1. Alexander DH, Novembre J, Lange K. Fast model-based estimation of ancestry in unrelated individuals. Genome Res. 2009;19: 1655–1664. doi:10.1101/gr.094052.109
