# Supplementary material for: A Prism Vote method for individualized risk prediction of traits in genotype data of Multi-population
Source: PLoS Genet. 2022 Oct 27;18(10):e1010443. doi: 10.1371/journal.pgen.1010443 (PMC9642904; doi:10.1371/journal.pgen.1010443)
Supplement: S7 Appendix — Fig A. PCA projections of the subjects from UK Biobank colored by inferred ancestry. S7 Appendix. Table A. Sample size of inferred ancestry populations in the UK Biobank data. S7 Appendix. Table B. Implementation of PV using LDpred2 as base model in the PAGE data. (DOCX) [file pgen.1010443.s007.docx]

# S7 Appendix. Implementing PV with summary statistics in mixed populations

To implement PV with summary statistics in mixed or admixed populations, ancestry-specific genetic effects shall be obtained in advance. We used the PAGE dataset to experiment PV’s application with the polygenetic risk score (PRS) in mixed populations. After dividing the original data into more homogeneous population strata, we next compute the PRS for each stratum, which is still composed of mixed or admixed subjects. To estimate PRS in mixed populations, we reference the method adopted by Ruan et al (2022) [1] that calculated a combined PRS by a weighted sum of ancestry-specific PRS. The summary statistics of single populations is obtained from the UK Biobank, in which the ancestral groups of subjects are assigned by PCA approach using the reference panel of 1000 Genomes Project, since the self-reported ancestral groups were vague or unspecific (**S7 Appendix. Figure A, S7 Appendix. Table A**) [2]. Next, we calculated the joint PRS in PAGE data based on the summary statistics of four ancestral groups in the UKB data, including the European (EUR), East Asian (EAS), African (AFR), and South Asian (SAS). The PRS methods adopted is the LDpred2 [3] computed by bigsnpr [4] in R. The combined PRS for stratum is calculated using a linear regression model with predictor variables including the four ancestry-specific PRSs, age, gender and top 10 PCs. Fitted coefficients from this model were used to derive the weighted PRS for predicting subjects in the test set. Prediction accuracy is obtained from 5GCV (**Materials and Methods**).

## S7 Appendix. Fig A. PCA projections of the subjects from UK Biobank colored by inferred ancestry

**
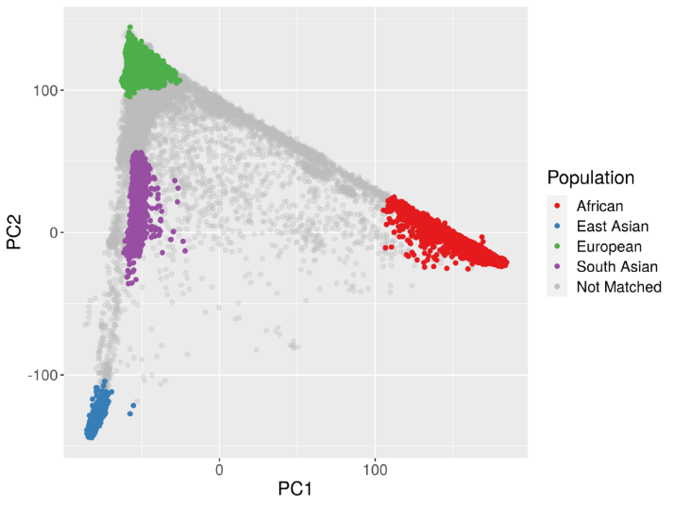
**

**Legend:** UK Biobank subjects are projected onto the PCA space calculated from the 1000 Genomes Project data, and are labeled by the inferred ancestry.

## S7 Appendix. Table A. Sample size of inferred ancestry populations in the UK Biobank data

| **Trait** | **AFR** | **EAS** | **EUR** | **SAS** |
| --- | --- | --- | --- | --- |
| **BMI** | 7,310 | 2,296 | 444,600 | 7,980 |
| **Height** | 7,322 | 2,299 | 445,074 | 7,989 |
| **Hypertension** | 7,428 (3,356/4,072)^*^ | 2,310 (616/1,694) | 446,069(150,703/295,366) | 8,159 (3,319/4,840) |
| **Diabetes** | 7,038 (794/6244) | 2,245 (129/2,116) | 437,364(22,645/414,719) | 7,648 (1,468/6,180) |

* In parenthesis: sample sizes of cases /controls for the binary traits

## S7 Appendix. Table B. Implementation of PV using LDpred2 as base model in the PAGE data

| Data | Models | BMI | Height | Hypertension | Diabetes |
| --- | --- | --- | --- | --- | --- |
| Mixed populations | LDpred2 + PCs | 0.372  (0.013) | 0.745  (0.006) | 0.598  (0.007) | 0.616  (0.007) |
|  | LDpred2 + PV | 0.389  (0.015) | 0.746  (0.006) | 0.598  (0.007) | 0.614  (0.010) |
| Single populations | LDpred2+ PCs  (Hawaiian) | 0.271  (0.024) | 0.771  (0.028) | 0.623  (0.035) | 0.615  (0.032) |
|  | LDpred2 + PCs  (African) | 0.182  (0.025) | 0.652  (0.048) | 0.590  (0.029) | 0.568  (0.022) |
|  | LDpred2 + PCs  (Japanese) | 0.292  (0.027) | 0.748  (0.008) | 0.538  (0.019) | 0.528  (0.009) |

**Legend**: To implement PV in mixed population data, we fitted a $Y\sim{PRS}_{weighted}+age+gender+10PCs$ in each training stratum, in which the PRS is weighted from the summary statistics of four ancestral groups. For single population analysis, PRS for the Japanese population referenced summary statistics of the EAS, and for the African population using AFR. However, for the Hawaiian population, no summary statistics is available, thus PRS of EUR is constructed for reference.

# References

1. Ruan Y, Lin Y-F, Feng Y-CA, Chen C-Y, Lam M, Guo Z, et al. Improving polygenic prediction in ancestrally diverse populations. Nat Genet. 2022;54: 573–580. doi:10.1038/s41588-022-01054-7

2. Privé F, Aschard H, Carmi S, Folkersen L, Hoggart C, O’Reilly PF, et al. Portability of 245 polygenic scores when derived from the UK Biobank and applied to 9 ancestry groups from the same cohort. Am J Hum Genet. 2022;109: 12–23. doi:10.1016/j.ajhg.2021.11.008

3. Privé F, Arbel J, Vilhjálmsson BJ. LDpred2: better, faster, stronger. Bioinformatics. 2020;36: 5424–5431. doi:10.1093/bioinformatics/btaa1029

4. Privé F, Aschard H, Ziyatdinov A, Blum MGB. Efficient analysis of large-scale genome-wide data with two R packages: bigstatsr and bigsnpr. Bioinformatics. 2018;34: 2781–2787. doi:10.1093/bioinformatics/bty185
