# Supplementary material for: A Prism Vote method for individualized risk prediction of traits in genotype data of Multi-population
Source: PLoS Genet. 2022 Oct 27;18(10):e1010443. doi: 10.1371/journal.pgen.1010443 (PMC9642904; doi:10.1371/journal.pgen.1010443)
Supplement: S8 Appendix — Table A. PAGE data. S8 Appendix. Table B. UK Biobank data. (DOCX) [file pgen.1010443.s008.docx]

# S8 Appendix. Prediction accuracies in various train-test population combinations

## S8 Appendix. Table A. PAGE data

|  | **Train** | **Test** | **DPR+PC** | **DPR+PV** | ***p*-value (PV vs reference method)** | | |
| --- | --- | --- | --- | --- | --- | --- | --- |
|  |  |  |  |  | **(1) Train: mixed; Test: mixed** | **(2)  Train: single; Test: single** | **(3)  Train: mixed; Test: single** |
| **BMI** | **Mixed** | **Mixed** | **0.305  (0.012)** | **0.374  (0.004)** | **<0.001*** |  |  |
|  | Hawaiian | Hawaiian | 0.250 (0.014) | 0.254 (0.017) |  | 0.699 |  |
|  | Mixed | Hawaiian | 0.226 (0.013) | 0.279 (0.033) |  |  | 0.021* |
|  | Japanese | Japanese | 0.299 (0.022) | 0.297 (0.024) |  | 0.917 |  |
|  | Mixed | Japanese | 0.152 (0.025) | 0.283 (0.030) |  |  | <0.001* |
|  | African | African | 0.196 (0.018) | 0.193 (0.017) |  | 0.787 |  |
|  | Mixed | African | 0.117 (0.020) | 0.154 (0.019) |  |  | 0.017* |
| **Height** | **Mixed** | **Mixed** | **0.718 (0.016)** | **0.739 (0.007)** | **0.043*** |  |  |
|  | Hawaiian | Hawaiian | 0.756 (0.027) | 0.755 (0.027) |  | 0.934 |  |
|  | Mixed | Hawaiian | 0.736 (0.022) | 0.753 (0.017) |  |  | 0.211 |
|  | Japanese | Japanese | 0.750 (0.008) | 0.750 (0.008) |  | 0.887 |  |
|  | Mixed | Japanese | 0.729 (0.019) | 0.750 (0.015) |  |  | 0.092 |
|  | African | African | 0.654 (0.048) | 0.654 (0.047) |  | 0.992 |  |
|  | Mixed | African | 0.624 (0.026) | 0.654 (0.017) |  |  | 0.071 |
| **Hypertension** | **Mixed** | **Mixed** | **0.544 (0.008)** | **0.589 (0.008)** | **<0.001*** |  |  |
|  | Hawaiian | Hawaiian | 0.612 (0.029) | 0.607 (0.027) |  | 0.812 |  |
|  | Mixed | Hawaiian | 0.560 (0.018) | 0.619 (0.034) |  |  | 0.133 |
|  | Japanese | Japanese | 0.546 (0.019) | 0.532 (0.034) |  | 0.463 |  |
|  | Mixed | Japanese | 0.526 (0.012) | 0.549 (0.021) |  |  | 0.241 |
|  | African | African | 0.592 (0.029) | 0.590 (0.030) |  | 0.920 |  |
|  | Mixed | African | 0.538 (0.008) | 0.591 (0.021) |  |  | 0.14 |
| **Diabetes** | **Mixed** | **Mixed** | **0.555 (0.011)** | **0.612 (0.022)** | **0.002*** |  |  |
|  | Hawaiian | Hawaiian | 0.602 (0.037) | 0.603 (0.037) |  | 0.964 |  |
|  | Mixed | Hawaiian | 0.568 (0.048) | 0.607 (0.041) |  |  | 0.192 |
|  | Japanese | Japanese | 0.555 (0.008) | 0.547 (0.005) |  | 0.089 |  |
|  | Mixed | Japanese | 0.524 (0.022) | 0.551 (0.032) |  |  | 0.227 |
|  | African | African | 0.581 (0.022) | 0.578 (0.021) |  | 0.839 |  |
|  | Mixed | African | 0.520 (0.019) | 0.577 (0.028) |  |  | 0.125 |

**p*-value <0.05

## S8 Appendix. Table B. UK Biobank data

|  | **Train** | **Test** | **DPR+PC^1^** | **DPR+PV^2^** | ***p*-value (PV vs reference method)** | | |
| --- | --- | --- | --- | --- | --- | --- | --- |
|  |  |  |  |  | **(1) Train: mixed; Test: mixed** | **(2) Train: single; Test: single** | **(3)  Train: mixed; Test: single** |
| **BMI** | **Mixed** | **Mixed** | **0.242  (0.007)** | **0.329  (0.014)** | **<0.001*** |  |  |
|  | Chinese | Chinese | 0.264 (0.038) | 0.264 (0.039) |  | 0.987 |  |
|  | Mixed | Chinese | 0.062 (0.026) | 0.035 (0.038) |  |  | 0.223 |
|  | Indian | Indian | 0.132 (0.030) | 0.132 (0.032) |  | 1 |  |
|  | Mixed | Indian | 0.114 (0.026) | 0.139 (0.021) |  |  | 0.140 |
|  | African | African | 0.335 (0.041) | 0.335 (0.040) |  | 1 |  |
|  | Mixed | African | 0.139 (0.029) | 0.294 (0.029) |  |  | <0.001* |
| **Height** | **Mixed** | **Mixed** | **0.728 (0.008)** | **0.753 (0.007)** | **<0.001*** |  |  |
|  | Chinese | Chinese | 0.725 (0.021) | 0.725 (0.021) |  | 0.977 |  |
|  | Mixed | Chinese | 0.704 (0.019) | 0.730 (0.012) |  |  | 0.037* |
|  | Indian | Indian | 0.774 (0.013) | 0.772 (0.013) |  | 0.827 |  |
|  | Mixed | Indian | 0.749 (0.017) | 0.778 (0.015) |  |  | 0.022* |
|  | African | African | 0.700 (0.016) | 0.700 (0.016) |  | 0.968 |  |
|  | Mixed | African | 0.666 (0.027) | 0.695 (0.021) |  |  | 0.096 |
| **Cardiovascular disease** | **Mixed** | **Mixed** | **0.646  (0.004)** | **0.751  (0.005)** | **<0.001*** |  |  |
|  | Chinese | Chinese | 0.769 (0.047) | 0.763 (0.048) |  | 0.857 |  |
|  | Mixed | Chinese | 0.644 (0.111) | 0.774 (0.081) |  |  | 0.069 |
|  | Indian | Indian | 0.753 (0.013) | 0.752 (0.012) |  | 0.960 |  |
|  | Mixed | Indian | 0.634 (0.018) | 0.749 (0.008) |  |  | <0.001* |
|  | African | African | 0.707 (0.029) | 0.710 (0.027) |  | 0.897 |  |
|  | Mixed | African | 0.615 (0.019) | 0.696 (0.022) |  |  | <0.001* |
| **Diabetes** | **Mixed** | **Mixed** | **0.641  (0.003)** | **0.716  (0.007)** | **<0.001*** |  |  |
|  | Chinese | Chinese | 0.651 (0.026) | 0.659 (0.031) |  | 0.662 |  |
|  | Mixed | Chinese | 0.626 (0.100) | 0.682 (0.059) |  |  | 0.321 |
|  | Indian | Indian | 0.679 (0.027) | 0.678 (0.027) |  | 0.936 |  |
|  | Mixed | Indian | 0.614 (0.005) | 0.683 (0.010) |  |  | <0.001* |
|  | African | African | 0.639 (0.024) | 0.641 (0.027) |  | 0.923 |  |
|  | Mixed | African | 0.613 (0.053) | 0.646 (0.036) |  |  | 0.291 |

**p*-value <0.05
