# Supplementary material for: A Prism Vote method for individualized risk prediction of traits in genotype data of Multi-population
Source: PLoS Genet. 2022 Oct 27;18(10):e1010443. doi: 10.1371/journal.pgen.1010443 (PMC9642904; doi:10.1371/journal.pgen.1010443)
Supplement: S1 Fig — (DOCX) [file pgen.1010443.s009.docx]

# S1 Fig. The genetic ancestries in simulation study I


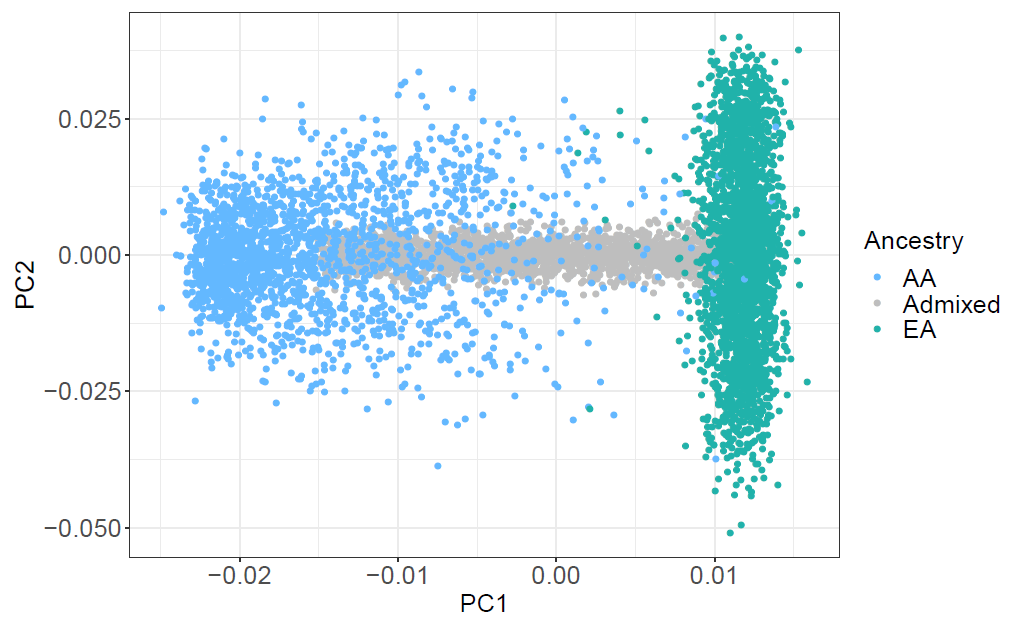


**Legend**: The three populations (European ancestry (EA), African ancestry (AA), Admixed population (Admixed) plotted in the coordinates spanned by the top two principal components (PCs).
