# Supplementary material for: A Prism Vote method for individualized risk prediction of traits in genotype data of Multi-population
Source: PLoS Genet. 2022 Oct 27;18(10):e1010443. doi: 10.1371/journal.pgen.1010443 (PMC9642904; doi:10.1371/journal.pgen.1010443)
Supplement: S1 Table — (DOCX) [file pgen.1010443.s010.docx]

# S1 Table. Prediction performance of the PV in real data application (PAGE dataset)

| Data | Models | BMI | Height | Hypertension | Diabetes |
| --- | --- | --- | --- | --- | --- |
| Mixed populations | DPR + PCs | 0.305^1^  (0.012) | 0.718  (0.016) | 0.544  (0.008) | 0.555  (0.011) |
|  | DPR + PV  (same No. of SNPs as DPR)^2^ | 0.342  (0.009) | 0.732  (0.007) | 0.572  (0.006) | 0.585  (0.022) |
|  | DPR + PV  (all SNPs) | 0.374  (0.004) | 0.739  (0.007) | 0.589  (0.008) | 0.612  (0.022) |
| Single populations | DPR + PCs  (Hawaiian, all SNPs) | 0.250  (0.014) | 0.756  (0.027) | 0.612  (0.029) | 0.602  (0.037) |
|  | DPR + PCs  (African, all SNPs) | 0.196  (0.018) | 0.654  (0.048) | 0.592  (0.029) | 0.581  (0.022) |
|  | DPR + PCs  (Japanese, all SNPs) | 0.299  (0.022) | 0.750  (0.008) | 0.546  (0.019) | 0.555  (0.008) |

^1^ The prediction accuracy for the continuous trait BMI and Height is evaluated by Pearson correlation coefficient in 5GCV, and for the dichotomous trait hypertension and diabetes by area-under-the-curve (AUC) in 5-group-cross-validation (5GCV). Standard deviation (SD) is given in parenthesis.

^2^ In mixed population analysis, we present two applications of DPR+PV, one using the same number of SNPs as the reference method (SNPs with *p*-value < 0.1), the other using all SNPs. This is because computing burden of DPR prevents its application in the entire data including all SNPs in mixed populations, while the framework of PV enables the DPR to be carried out with all SNPs within stratum. In single population analysis, DPR is calculated including all SNPs.
