# Supplementary material for: A Prism Vote method for individualized risk prediction of traits in genotype data of Multi-population
Source: PLoS Genet. 2022 Oct 27;18(10):e1010443. doi: 10.1371/journal.pgen.1010443 (PMC9642904; doi:10.1371/journal.pgen.1010443)
Supplement: S2 Table — (DOCX) [file pgen.1010443.s011.docx]

# S2 Table. Sample size of non-European populations in the UK Biobank

| **Populations** | **Sample size** |
| --- | --- |
| Indian | 5716 |
| Other ethnic group | 4356 |
| Caribbean | 4297 |
| African | 3204 |
| Pakistani | 1748 |
| Any other Asian background | 1747 |
| Chinese | 1504 |
| Any other mixed background | 996 |
| White and Asian | 802 |
| White and Black Caribbean | 597 |
| NA | 522 |
| White and Black African | 402 |
| Bangladeshi | 221 |
| Do not know | 204 |
| Any other Black background | 118 |
| Mixed | 46 |
| Black or Black British | 26 |
